# Prevalence of microhematuria in renal colic and urolithiasis: a systematic review and meta-analysis

**DOI:** 10.1186/s12894-020-00690-7

**Published:** 2020-08-08

**Authors:** Bruno Minotti, Giorgio Treglia, Mariarosa Pascale, Samuele Ceruti, Laura Cantini, Luciano Anselmi, Andrea Saporito

**Affiliations:** 1 Emergency Department, Cantonal Hospital of St. Gallen, Rorschacher Strasse 95, CH-9007 St. Gallen, Switzerland; 2 Heath Technology Assessment Unit, Ente Ospedaliero Cantonale, Bellinzona, Switzerland; 3 Clinical Trial Unit, Ente Ospedaliero Cantonale, Bellinzona, Switzerland; 4 Department of Intensive Care Medicine, Clinica Luganese, Lugano, Switzerland; 5 Department of Anesthesia, Ospedale San Giovanni, Bellinzona, Switzerland

**Keywords:** Renal colic, Urolithiasis, Microhematuria, Stone score

## Abstract

**Background:**

This systematic review and meta-analysis aims to investigate the prevalence of microhematuria in patients presenting with suspected acute renal colic and/or confirmed urolithiasis at the emergency department.

**Methods:**

A comprehensive literature search was conducted to find relevant data on prevalence of microhematuria in patients with suspected acute renal colic and/or confirmed urolithiasis. Data from each study regarding study design, patient characteristics and prevalence of microhematuria were retrieved. A random effect-model was used for the pooled analyses.

**Results:**

Forty-nine articles including 15′860 patients were selected through the literature search. The pooled microhematuria prevalence was 77% (95%CI: 73–80%) and 84% (95%CI: 80–87%) for suspected acute renal colic and confirmed urolithiasis, respectively. This proportion was much higher when the dipstick was used as diagnostic test (80 and 90% for acute renal colic and urolithiasis, respectively) compared to the microscopic urinalysis (74 and 78% for acute renal colic and urolithiasis, respectively).

**Conclusions:**

This meta-analysis revealed a high prevalence of microhematuria in patients with acute renal colic (77%), including those with confirmed urolithiasis (84%). Intending this prevalence as sensitivity, we reached moderate values, which make microhematuria alone a poor diagnostic test for acute renal colic or urolithiasis. Microhematuria could possibly still important to assess the risk in patients with renal colic.

## Background

Renal colic is caused by the presence of stones in the urinary tract and it is characterized by sudden onset of severe loin pain, radiating to the flank, groin, and testes or *labia majora* [1]. Incidence amounts to 240 per 100′000 persons [2] with a prevalence up to 10%; men are commonly more affected than women with a ratio of 3–2:1 [3]. Lifetime risk is up to 19% in men and 9% in women [4], varying depending on geographic location and increasing constantly over last years [5]. Guidelines for the diagnostic pathway suggest assessing (micro) hematuria, while the gold standard of imaging is unenhanced multi-detector computed tomography (MDCT) [1]. As diagnostic tool the STONE Score was developed and validated; this score includes parameters as sex, duration of pain prior to presentation, race, nausea, vomiting and microhematuria [6]. Microhematuria prevalence in suspected renal colic has been studied in several trials, ranging from 55% [7] to 93% [8, 9]. In order to better understand the difference existing in prevalence range, we performed a meta-analysis of studies dealing with microhematuria by suspected acute renal colic and/or confirmed urolithiasis.

## Methods

This systematic review and meta-analysis conforms to the statement on Preferred Reporting Items for Systematic reviews and Meta-Analyses [10].

### Search strategy

A literature search of the electronic PubMed/MEDLINE database and Cochrane Central Register of Controlled Trials (CENTRAL), without language restriction, was carried out from inception to October 11, 2018. A search algorithm was established using a combination of the following terms: A) renal colic AND urolithiasis (Problem), B) urinalysis (Intervention), C) microhematuria (Outcome). The final search query is reported in Appendix 1. Reference lists of the retrieved articles were also screened for additional studies.

### Eligibility criteria

We included in this systematic review and meta-analysis studies which filled the following inclusion criteria: a) original article published in peer-reviewed journal; b) studies including adults only; c) patients presenting with acute renal colic at the emergency department; d) studies reporting data on microhematuria.

Exclusion criteria were: a) articles not within the field of interest of this review; b) review articles, letters or editorials; c) case reports or case series (less than 10 patients included); d) articles with possible patient data overlap.

### Study selection

Titles and abstracts of the retrieved studies were independently reviewed by two researchers (MP, GT), applying the inclusion and exclusion criteria mentioned above. Articles were rejected if they were clearly ineligible. The full texts of the potentially eligible articles were reviewed independently by the same researchers to confirm or exclude their eligibility for inclusion. Disagreements were resolved in a consensus meeting.

### Data extraction

For each included study, one author (MP) manually extracted data relevant to the review aims using a customized form. Information regarding basic study data (authors, year of publication, country of origin, type of study), patient characteristics (number of patients, mean age, gender), methods (microhematuria test, microhematuria definition) and outcomes (number of patients with microhematuria, microhematuria prevalence) were retrieved. The number of patients with microhematuria and microhematuria prevalence were also extracted for patients with confirmed urolithiasis, where available. Diagnostic methods for detection of stones were also retrieved. One other author (GT) independently checked all extracted data.

### Outcome measures

The primary outcome was the percentage of microhematuria among patients presenting with suspected acute renal colic at the emergency department. The secondary outcome was the percentage of microhematuria among patients presenting with acute renal colic and confirmed urolithiasis at the emergency department.

### Quality assessment

The overall quality of the studies included in the systematic review was critically appraised based on the revised “Quality Assessment of Diagnostic Accuracy Studies” tool (QUADAS-2). This tool comprises four domains: patient selection, index test, reference standard, and flow and timing. Each domain was assessed in terms of risk of bias, and the first three domains were also assessed in terms of concerns regarding applicability. Two authors have performed the risk of bias assessment (GT and MP) reaching a consensus.

### Statistical analysis

Microhematuria prevalence was defined as the ratio between the number of patients with suspected acute renal colic with microhematuria detected by urinalysis or dipstick and the total number of patients with suspected acute renal colic who underwent the analysis. This proportion was calculated also for patients presenting with acute renal colic and confirmed urolithiasis.

Pooled analyses of the proportion of microhematuria detected by urinalysis or dipstick were performed using data retrieved from the selected studies. When microhematuria was assessed using both urinalysis and dipstick, the test with the better outcome was chosen. Subgroup analyses taking into account the microhematuria test were planned.

A random-effects model was used for statistical pooling of the data, taking into account the heterogeneity between studies. The different weight of each study in the pooled analysis was related to the different sample size. Pooled data were presented with their respective 95% confidence interval (95%CI) values, and data were displayed using plots.

Heterogeneity was estimated by using the I-square index (I^2^), which describes the percentage of variation across studies that is due to heterogeneity rather than chance [11] and considered significant if I-square test was higher than 50%.

Publication bias was assessed through the Egger’s test [12].

Statistical analyses were performed using the StatsDirect software version 3 (StatsDirect Ltd., Cambridge, UK).

## Results

### Literature search

**Fig. 1:**
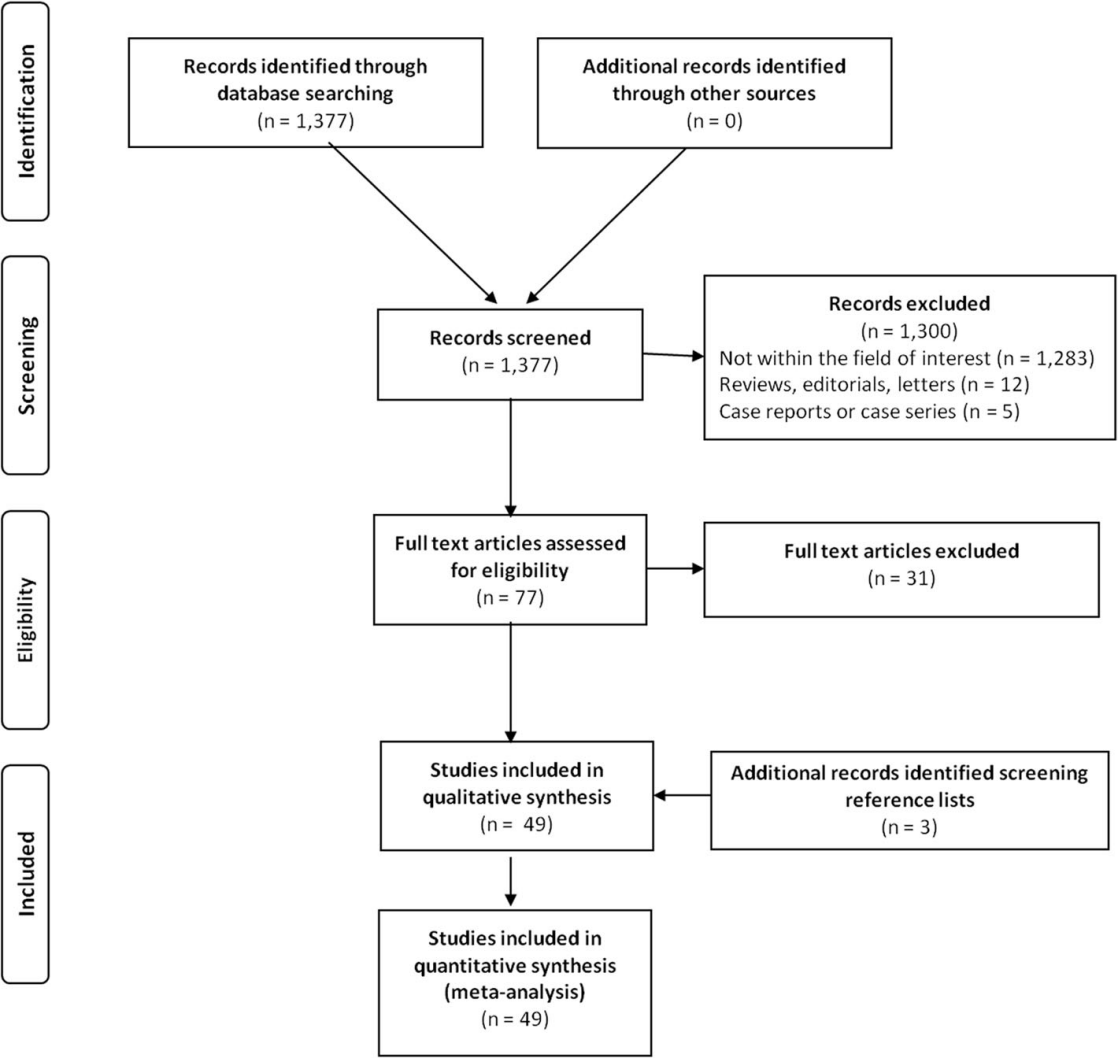
PRISMA flow chart of the retrieved, excluded and analyzed studies

The literature search from PubMed/MEDLINE and Cochrane CENTRAL databases yielded a total of 1377 records. After reviewing titles and abstracts, 77 were selected as potentially eligible articles. The full text was retrieved for all. Following eligibility’s assessment, 31 articles did not meet the inclusion criteria and were excluded from the systematic review. Within the selected articles, screening of the reference lists allowed to add 3 additional records. Finally, 49 studies [7–9, 13–58] including 15′860 patients were identified as potentially relevant and were selected for the systematic review and meta-analysis. All of the included studies except two [30, 50] were published in English. These studies covered the period from inception to October 11, 2018. Search results and articles’ selection are displayed in a PRISMA flow chart (Fig. 1).

### Selected studies

The characteristics of selected studies are reported in Table 1. The studies were conducted in different countries worldwide (Europe, North America, Asia, Africa). The sample size of the included trials ranged from 32 to 2218 adults presenting to the emergency department or urology clinic with acute renal colic. Most of the studies were observational with a prospective (19) or retrospective (29) or mixed (1) design.

**Table 1:** Basic study and patient characteristics. Patients presenting with acute renal colic at the emergency department

Authors	Year	Country	Study design	No. of patients	% Male	Mean age ± SD (years)
Kim et al. [13]	2018	South Korea	Retrospective, observational	798	68.6	48.2 ± 13.3
Desai et al. [14]	2018	USA	Retrospective, observational	350	NR	NR
Türk *and* Ün [15]^a^	2017	Turkey	Prospective, observational	516	60.5	37 ± 20.3
Shrestha et al. [16]^a^	2017	Nepal	Retrospective, observational	201	55.2	29 ± 13.5
Odoemene et al. [17]^a^	2017	Nigeria	Prospective, observational	69	76.8	40.4 ± 2.9
Mefford et al. [18]	2017	USA	Retrospective, observational	393	69	Median 43 (IQR 32–54)
Rapp et al. [19]	2016	USA	Retrospective, observational	613	47	49 ± 0.6
Park et al. [20]	2016	South Korea	Prospective, RCT	103	66	45.6 ± 12.55
Hernandez et al. [21]	2016	USA	Retrospective, observational	536	56	45.9 ± 16.3
Fukuhara et al. [22]^a^	2016	Japan	Retrospective, observational	491	70.5	51.8 ± 15
Dorfman et al. [23]	2016	USA	Retrospective, observational	339	55.5	46.8 ± 16.5
Yan et al. [24]	2015	Canada	Prospective cohort study	565	62.8	46.6 ± 14.4
Lee et al. [25]	2015	South Korea	Retrospective, observational	2218	71	43.3 ± 14.2
Hall et al. [26]^a^	2015	UK	Retrospective, observational	513	57.1	45 ± 23.3
Zwank et al. [27]	2014	USA	Prospective, observational	93	NR	39 ± NR
Abdel-Gawad et al. [28]^a^	2014	UAE	Retrospective, observational	939	87.9	37.9 ± 11
Inci et al. [7]	2013	Turkey	Retrospective, observational	83	42.2	42.1 ± 14.4
Lallas et al. [29]	2011	USA	Prospective, observational	32	NR	NR
Perez et al. [30]^a^	2010	Spain	Prospective, multicentre, cross-sectional case-control	146	57.53	51.34 ± NR
Xafis et al. [31]^a^	2008	Switzerland	Retrospective, observational	638	NR	44.3 ± 14.6
Serinken et al. [32]^a^	2008	Turkey	Retrospective, observational	235	75.7	31.1 ± 7
Cupisti et al. [33]	2008	Italy	Retrospective, observational	696	54	NR
Matani *and* Al-Ghazo [34]^a^	2007	Saudi Arabia / Jordan	Retrospective, observational	75	61.3	42.2 ± NR
Kartal et al. [35]^a^	2006	Turkey	Prospective, observational	227	64.8	38.4 ± 14
Kirpalani et al. [36]	2005	Canada	Retrospective, observational	299	NR	NR
Gaspari *and* Horst [37]	2005	USA	Prospective, observational	110	NR	NR
Argyropoulos et al. [8]	2004	Greece	Retrospective, observational	609	63.2	49.2 ± 15.9
Unal et al. [38]^a^	2003	Turkey	Prospective, observational	137	55	38 ± NR
Tack et al. [39]^a^	2003	Belgium	Prospective, observational	106	50	45 ± NR
Kobayashi et al. [40]	2003	Japan	Retrospective, observational	537	78	46.6 ± 14
Eray et al. [41]	2003	Turkey	Prospective, observational	65	60	38.8 ± 13.5
Lucks et al. [42]	2002	USA	Retrospective, observational	587	NR	NR
Hamm et al. [43]	2002	Germany	Prospective, observational	109	69.7	49 ± NR
Li et al. [44]^a^	2001	USA	Retrospective, observational	397	73	47 ± 15
Hamm et al. [45]	2001	Germany	Prospective, observational	125	72	55 ± 17
Richards *and* Christman [46]	1999	USA	Retrospective, observational	185	NR	NR
Bove et al. [47]	1999	USA	Retrospective, observational	195	NR	NR
Ooi et al. [9]^a^	1998	Singapore	Prospective, observational	122	93	39.7 ± NR
Ghali et al. [48]^a^	1998	Saudi Arabia	Prospective, observational	125	80	39.2 ± NR
Eskelinen et al. [49]	1998	Finland	Prospective, observational	57	NR	NR
Gimondo et al. [50]^a^	1996	Italy	Retrospective, observational	76	60.5	47 ± NR
Boyd *and* Gray [51]	1996	UK	Prospective, observational	52	NR	NR
Press *and* Smith [52]	1995	USA	Retrospective, observational	109	NR	NR
Chia et al. [53]	1995	Singapore	Prospective, observational	294	72.5	43.5 ± NR
Elton et al. [54]^a^	1993	USA	Retrospective / prospective, observational	275	71.2	46.2 ± 15.7
Stewart et al. [55]	1990	USA	Retrospective, observational	160	76.9	NR
Freeland [56]	1987	Northern Ireland	Retrospective, observational	134	NR	NR
Dunn et al. [57]	1985	USA	Retrospective, observational	76	NR	42.7 ± NR
Bishop [58]	1980	UK	Prospective, observational	50	NR	NR

*Abbreviations* (alphabetical order): *IQR* interquartile range, *NR* not reported, *RCT* Randomized controlled study, *SD* standard deviation, *UAE* United Arab Emirates, *UK* United Kingdom, *USA* United States of America

^a^Enrolled also children

Microhematuria was tested by urinalysis in 32 studies, urine dipstick in 10 and both methods in 7. Definition of microhematuria was different among the included studies. Six studies included also patients presenting with macroscopic hematuria [14, 17, 19, 22, 26, 50]. Details on the microhematuria test are reported in Table 2.

**Table 2:** Data on microhematuria in patients presenting with suspected acute renal colic at the emergency department

Authors	Microhematuria test	Type of hematuria	Positive microhematuria definition	No. patients with microhematuria	Microhematuria prevalence
Kim et al. [13]	Urinalysis	Microscopic	Presence of 4 or more RBCs/HPF	750	750/798 (94%)
Desai et al. [14]	Urinalysis	Microscopic or macroscopic	Positive urinalysis for RBCs or for blood	245	245/350 (70%)
Türk *and* Ün [15]	Urinalysis	Microscopic	NR	432	432/516 (83.7%)
Shrestha et al. [16]	Urinalysis	Microscopic	Presence of 3 or more RBCs	70	70/201 (34.8%)
Odoemene et al. [17]	Urinalysis	Microscopic or macroscopic	NR	62	62/69 (89.9%)
Mefford et al. [18]	Urinalysis	Microscopic	Presence of 4 or more RBCs/HPF	321	321/393 (81.7%)
Rapp et al. [19]	Urinalysis	Microscopic or macroscopic	Presence of 4 or more RBCs/HPF	412	412/613 (67.2%)
Park et al. [20]	Urinalysis	Microscopic	NR	90	90/103 (87.4%)
Hernandez et al. [21]	Urine dipstick	Microscopic	Hematuria on urine dipstick	332	332/536 (61.9%)
Fukuhara et al. [22]	Urinalysis or urine dipstick	Microscopic or macroscopic	Occult blood in urine	352	352/491 (71.7%)
Dorfman et al. [23]	Urinalysis	Microscopic	Presence of 5 or more RBCs/HPF	254	254/339 (74.9%)
Yan et al. [24]	Urinalysis	Microscopic	NR	451	451/565 (79.8%)
Lee et al. [25]	Urinalysis	Microscopic	NR	1980	1980/2218 (89.3%)
Hall et al. [26]	Urine dipstick	Microscopic or macroscopic	Scores of 1+ to 3+ on urine dipstick or documented frank hematuria	391	391/513 (76.2%)
Zwank et al. [27]	Urinalysis	Microscopic	RBCs present	66	66/93 (71%)
Abdel-Gawad et al. [28]	Urinalysis	Microscopic	Presence of 4 or more RBCs/HPF	835	835/939 (88.9%)
Inci et al. [7]	Urinalysis	Microscopic	Presence of 5 or more RBCs/HPF	46	46/83 (55.4%)
Lallas et al. [29]	Urinalysis	Microscopic	Presence of 4 or more RBCs/HPF	18	18/32 (56.3%)
	Urine dipstick	Microscopic	Trace or scores of 1+ to 4+ on urine dipstick	21	21/32 (65.6%)
Perez et al. [30]	Urine dipstick	Microscopic	NR	132	132/146 (90.4%)
Xafis et al. [31]	Urinalysis	Microscopic	Presence of 5 or more RBCs/HPF	396	396/638 (62.1%)
Serinken et al. [32]	Urinalysis	Microscopic	Presence of 5 or more RBCs/HPF	194	194/235 (82.6%)
Cupisti et al. [33]	Urine dipstick	Microscopic	NR	592	592/696 (85.1%)
Matani *and* Al-Ghazo [34]	Urinalysis	Microscopic	Presence of 4 or more RBCs/HPF	50	50/75 (66.7%)
Kartal et al. [35]	Urinalysis	Microscopic	Presence of 10 or more RBCs/HPF	146	146/227 (64.3%)
Kirpalani et al. [36]	Urine dipstick	Microscopic	Positive urine dipstick	228	228/299 (76.3%)
Gaspari *and* Horst [37]	Urinalysis	Microscopic	Presence of 5 or more RBCs/HPF	82	82/110 (74.5%)
Argyropoulos et al. [8]	Urine dipstick	Microscopic	Scores of 1+ to 3+ on urine dipstick	566	566/609 (92.9%)
Unal et al. [38]	Urinalysis	Microscopic	Presence of 4 or more RBCs/HPF	100	100/137 (73%)
Tack et al. [39]	Urinalysis or Urine dipstick	Microscopic	Presence of 2 or more RBCs/HPF or positive dipstick	77	77/106 (72.6%)
Kobayashi et al. [40]	Urine dipstick	Microscopic	Scores of 1+ to 3+ on urine dipstick	382	382/537 (71.1%)
	Urinalysis	Microscopic	Presence of 5 or more RBCs/HPF	350	350/537 (65.2%)
Eray et al. [41]	Urinalysis	Microscopic	Presence of 6 or more RBCs/HPF	45	45/20 (69.2%)
Luchs et al. [42]	Urinalysis	Microscopic	Presence of 10 or more RBCs/HPF	492	492/587 (83.8%)
Hamm et al. [45]	Urinalysis	Microscopic	Presence of more than 20 mg/dl hemoglobin	66	66/109 (60.6%)
Li et al. [44]	Urinalysis or Urine dipstick	Microscopic	Presence of any number of RBCs/HPF or trace / scores of 1+ to 3+ on urine dipstick	360	360/397 (90.7%)
Hamm et al. [45]	Urinalysis	Microscopic	Presence of 4 or more RBCs/HPF	99	99/125 (79.2%)
Richards *and* Christman [46]	Urinalysis	Microscopic	Presence of 4 or more RBCs/HPF	156	156/185 (84.3%)
Bove et al. [47]	Urine dipstick	Microscopic	Positive urine dipstick	130	130/180 (72.2%)
	Urinalysis	Microscopic	Presence of 6 or more RBCs/HPF	128	128/195 (65.6%)
	Urinalysis or Urine dipstick	Microscopic	Presence of 2 or more RBCs/HPF or positive urine dipstick	153	153/195 (78.5%)
Ooi et al. [9]	Urine dipstick	Microscopic	Scores of 1+ or more on urine dipstick	114	114/122 (93.4%)
	Urinalysis	Microscopic	Presence of 6 or more RBCs/HPF in males or of 10 or more RBCs/HPF in females	77	77/122 (63.1%)
Ghali et al. [48]	Urinalysis	Microscopic	Presence of 4 or more RBCs/HPF	81	81/125 (64.8%)
Eskelinen et al. [49]	Urinalysis	Microscopic	Presence of 11 or more RBCs/HPF	43	43/57 (75.4%)
Gimondo et al. [50]	Urine dipstick	Microscopic or macroscopic	Positive urine dipstick	56	56/76 (73.7%)
Boyd *and* Gray [51]	Urine dipstick	Microscopic	Positive urine dipstick	45	45/52 (86.5%)
Press *and* Smith [52]	Urinalysis	Microscopic	Presence of 1 or more RBCs/HPF	78	78/109 (71.6%)
Chia et al. [53]	Urinalysis	Microscopic	Presence of 6 or more RBCs/HPF in males or of 10 or more RBCs/HPF in females	181	181/294 (61.6%)
Elton et al. [54]	Urinalysis	Microscopic	Presence of 4 or more RBCs/HPF	194	194/275 (70.5%)
Stewart et al. [55]	Urinalysis	Microscopic	Presence of 3 or more RBCs/HPF	132	132/160 (82.5%)
Freeland [56]	Urine dipstick	Microscopic	Trace or scores of 1+ to 3+ on urine dipstick	102	102/134 (76.1%)
Dunn et al. [57]	Urinalysis	Microscopic	Presence of 3 or more RBCs/HPF	62	62/76 (81.6%)
Bishop [58]	Urine dipstick	Microscopic	Positive urine dipstick	44	44/50 (88%)

*Abbreviations* (alphabetical order): *NR* not reported, *HPF* High power Field, *RBC* Red Blood Cell

### Quality assessment

Overall quality assessment of the studies included in the systematic review according to QUADAS-2 tool is reported in Supplemental Figure 1.

### Microhematuria prevalence and suspected acute renal colic

**Fig. 2:**
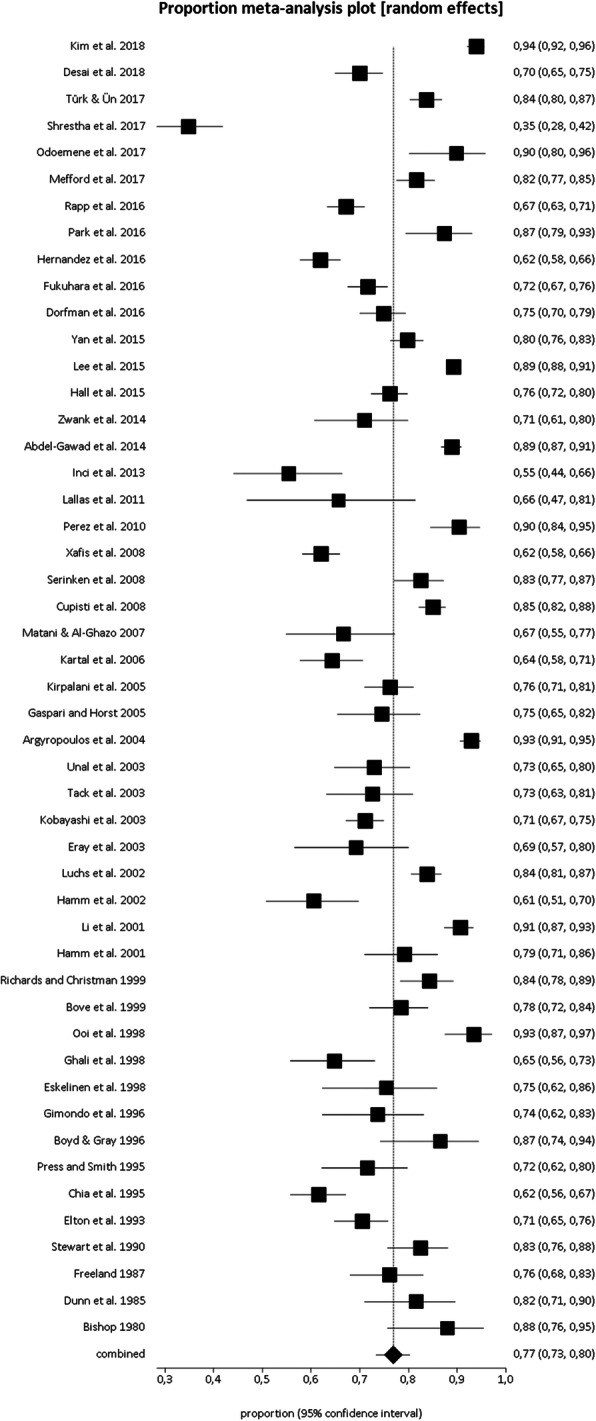
Plots of individual studies and pooled prevalence of microhematuria in patients with acute renal colic, including 95% confidence intervals (95%CI)

Primary outcome characteristics on microhematuria prevalence in patients with suspected acute renal colic are summarized in Table 2 and Fig. 2.

Prevalence of microhematuria ranged from 35 to 94%, with a pooled estimate of 77% (95%CI: 73–80%) (Fig. 2). The heterogeneity among the included studies was significant (I^2^ = 96%). A publication bias was detected by Egger’s test (*p* < 0.0001).

Performing sub-group analyses taking into account different microhematuria tests, the pooled prevalence of microhematuria using urinalysis or urine dipstick was 74% (95%CI: 69–78%) and 80% (95%CI: 74–86%) respectively, without significant difference between two groups.

### Microhematuria prevalence and confirmed urolithiasis

**Fig. 3:**
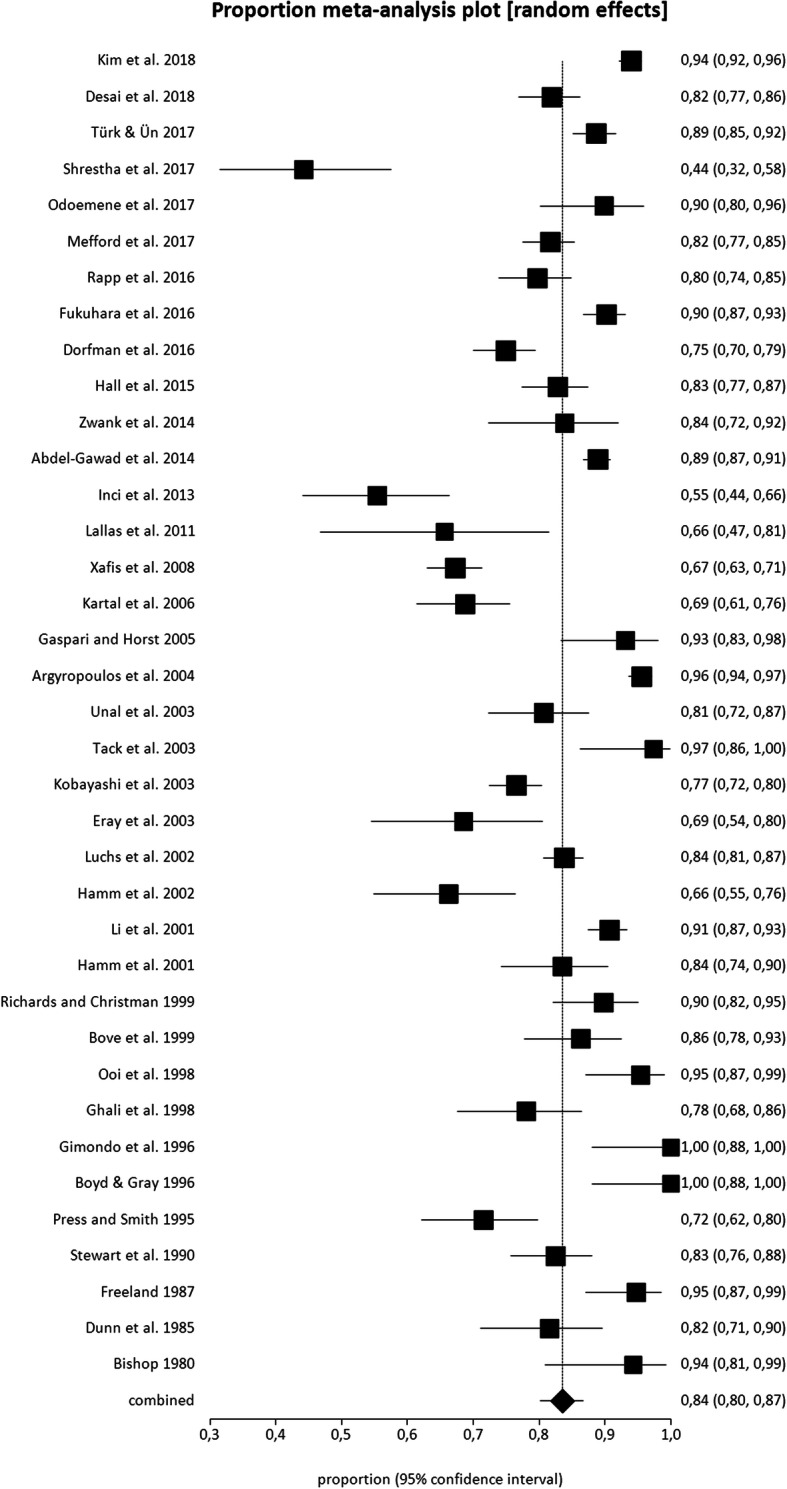
Plots of individual studies and pooled prevalence of microhematuria in patients with confirmed urolithiasis, including 95% confidence intervals (95%CI)

Secondary outcomes regarding main findings on microhematuria prevalence in patients with acute renal colic and confirmed urolithiasis are summarized in Table 3 and Fig. 3.

**Table 3:** Data on microhematuria in patients presenting with confirmed urolithiasis at the emergency department

Authors	Microhematuria test	No. patients with microhematuria	Microhematuria prevalence	Diagnostic test for urolithiasis
Kim et al. [13]	Urinalysis	750	750/798 (94%)	Unenhanced MDCT
Desai et al. [14]	Urinalysis	231	231/282 (81.9%)	Non-contrast CT
Türk et al. [15]	Urinalysis	344	344/388 (88.7%)	Non-contrast complete abdominal CT
Shrestha et al. [16]	Urinalysis	27	27/61 (44.3%)	Renal US
Odoemene et al. [17]^a^	Urinalysis	62	62/69 (89.9%)	Abdominal US, IVU, CT
Mefford et al. [18]	Urinalysis	321	321/393 (81.7%)	Non-contrast abdominal or pelvic CT
Rapp et al. [19]^a^	Urinalysis	177	177/222 (79.7%)	Non-contrast CT
Fukuhara et al. [22]^a^	Urinalysis or urine dipstick	323	323/358 (90.2%)	Plain abdominal X-ray, helical contrast enhanced or non-contrast CT
Dorfman et al. [23]	Urinalysis	254	245/339 (74.9%)	Abdominal CT
Hall et al. [26]^a^	Urine dipstick	193	193/233 (82.8)	Non-enhanced CT
Zwank et al. [27]	Urinalysis	52	52/62 (83.9)	CT
Abdel-Gawad et al. [28]	Urinalysis	835	835/939 (88.9)	Color doppler or gray-scale US, abdomen X-ray, helical CT
Inci et al. [7]	Urinalysis	46	46/83 (55.4)	Unenhanced MDCT
Lallas et al. [29]	Urinalysis	18	18/32 (56.3)	US, Abdomen X-ray, IVU, CT
	Urine dipstick	21	21/32 (65.6)	
Xafis et al. [31]	Urinalysis	341	341/507 (67.3)	Unenhanced MDCT
Kartal et al. [35]	Urinalysis	121	121/176 (68.8)	IVU, US, spiral CT, stone passage
Gaspari *and* Horst [37]	Urinalysis	54	54/58 (93.1)	US, CT
Argyropoulos et al. [8]	Urine dipstick	539	539/564 (95.6)	Abdomen X-ray, US
Unal et al. [38]	Urinalysis	92	92/114 (80.7)	US, excretory urography, non-enhanced helical CT
Tack et al. [39]	Urinalysis or Urine dipstick	37	37/38 (97.4)	Excretory urography, non-enhanced helical MDCT
Kobayashi et al. [40]	Urine dipstick	346	346/452 (76.5)	Abdomen X-ray, US, CT
	Urinalysis	317	317/452 (70.1)	
Eray et al. [41]	Urinalysis	37	37/54 (68.5)	Abdomen X-ray, spiral CT, stone passage
Luchs et al. 42[]	Urinalysis	492	492/587 (83.8)	CT, stone passage
Hamm et al. [43]	Urinalysis	53	53/80 (66.3)	Unenhanced low dose elical CT
Li et al. [44]	Urinalysis or Urine dipstick	360	360/397 (90.7)	CT, IVP
Hamm et al. [45]	Urinalysis	76	76/91 (83.5)	Helical CT
Richards *and* Christman [46]	Urinalysis	88	88/98 (89.8)	IVU
Bove et al. [47]	Urine dipstick	70	70/87 (80.5)	CT
	Urinalysis	77	77/95 (81.1)	
	Urinalysis or Urine dipstick	82	82/95 (86.3)	
Ooi et al. [9]	Urine dipstick	62	62/65 (95.4)	Abdomen X-ray, IVU
	Urinalysis	46	46/65 (70.8)	
Ghali et al. [48]	Urinalysis	64	64/82 (78)	Abdomen X-ray, IVU, US
Gimondo et al. [50]^a^	Urine dipstick	29	29/29 (100)	US
Boyd *and* Gray [51]	Urine dipstick	29	29/29 (100)	Abdomen X-ray, IVU
Press *and* Smith [52]	Urinalysis	78	78/109 (71.6)	IVU
Stewart et al. [55]	Urinalysis	132	132/160 (82.5)	IVP
Freeland [56]	Urine dipstick	72	72/76 (94.7)	IVU or stone passage
Dunn et al. [57]	Urinalysis	62	62/76 (81.6)	IVU or stone passage
Bishop [58]	Urine dipstick	33	33/35 (94.3)	IVU

*Abbreviations* (alphabetical order): *CT* computed tomography, *HFU* High-power field, *IVU* Intravenous Urography, *MDCT* multidetector CT, *NR* not reported, *RBC* Red Blood Cell, *SD* standard deviation, *US* ultrasound

^a^This study included also patients with gross hematuria

Prevalence of microhematuria ranged from 44 to 100%, with a pooled estimate of 84% (95%CI: 80–87%) (Fig. 3). Heterogeneity among the included studies was significant (I^2^ = 93%). A publication bias was detected by Egger’s test (*p* = 0.0008).

Performing sub-group analyses taking into account different microhematuria tests, the pooled prevalence of microhematuria using urinalysis or urine dipstick was 78% (95%CI: 74–82%) and 90% (95%CI: 83–95%), respectively.

## Discussion

Many studies have evaluated the prevalence of microhematuria in patients with suspected acute renal colic (Table 1); this meta-analysis pooled data reported in the published studies to derive a more precise assessment. Overall, this systematic review and meta-analysis revealed a high prevalence of microhematuria in patients with acute renal colic (77%), including those with confirmed urolithiasis (84%). However, intending this prevalence as sensitivity, we reached moderate values, which make microhematuria alone a poor diagnostic test for acute renal colic, respectively for urolithiasis. In our meta-analysis heterogeneity was high; indeed, we found a poor definition regarding urine analysis across studies (see positive microhematuria definition in Table 2), with different cells count on microscopy, but also with various dipstick brands. Argyropoulos et al. [8] carried out a microscopic urinalysis when the dipstick was in doubt or with blood traces; microhematuria was confirmed in all of these cases. Thus, the authors concluded that urinary dipstick test is not inferior to microscopy. Bataille et al. [59] compared the sensitivity of urinary dipstick with microscopy and flow cytometry on in vitro contaminated human urine with human blood of volunteers at different concentrations. Urinary dipstick reached the best sensitivity, probably due to the ability to detect red blood cells after lysis, and was suggested as preferred test for screening of hematuria. Same results were previously reported by Kobayashi et al. [40] and Press et al. [52]. De facto we detected a trend toward a higher pooled prevalence of microhematuria by using urine dipstick compared to microscopic urinalysis. Some studies analyzed the characteristics of patients with renal colic and negative microhematuria, the most without correlation between size, location or composition of the stones, or grade of the obstruction [44, 52, 55, 57]. Kobayashi et al. [40] found a relation between hematuria and pain onset, with the highest incidence of negative hematuria on day 3 and 4. Kim et al. [13] found negative microhematuria in patients with lower stones or elevated serum blood urea nitrogen (BUN). Mefford et al. [18] showed an increased prevalence of hydronephrosis in patients with urolithiasis and negative microhematuria. As hydronephrosis is easy to screen with ultrasonography, Daniel et al. [60] developed the STONE PLUS Score with addition of point-of-care ultrasound of the kidney to the original STONE Score. Presence of hydronephrosis improved the specificity up to 98% and helped to identify patients requiring urological intervention, without remarkably increasing risk stratification.

Considering the moderate sensitivity of microhematuria in patients with renal colic, Xafis et al. [31] suggested to perform a MDCT without urinalysis as a prerequisite. This approach seems to show the best diagnostic accuracy; however, it would increase the number of MDCT with more costs and radiation exposure. Therefore, the focus should be placed in complicated urolithiasis (e.g., obstructive pyelonephritis) or dangerous alternative diagnosis. Rucker et al. [61] reported numerous diseases mimicking urolithiasis. Moore et al. [6] found a lower likelihood of a dangerous alternative diagnosis (< 2%) by using high STONE scores and suggested for this group the possibility to initially avoid compute tomography because till 90% of stones < 7 mm will pass through spontaneously [62]. With the same approach the American College of Emergency Physicians (ACEP) suggests in the Choosing Wisely group to avoid ordering computed tomography of the abdomen and pelvis in young except healthy emergency department patients (age < 50) with known histories of kidney stones, or ureterolithiasis, presenting with symptoms consistent with uncomplicated renal colic [63]. In fact, taking all studies together, the prevalence of patients with renal colic having effectively urolithiasis was 66% (median, IQR 52–76), which means a higher pre-test probability in the studied population and so a good discerning capacity of the treating physicians. Anyway, alternative diagnoses mimicking renal colic have to be taken into account. Commons diagnoses are pyelonephritis, appendicitis, diverticulitis, adnexal cysts/tumor, cholecystitis, and lumbago/sciatica. Rarer pneumonia, lymphoma or aortic dissection/aneurysm. However CT scan negative rate reach till 31% [42] and Zwank et al. [27] could show that CT scan didn’t change management when providers did not expect it would. Finally, alternative diagnosis mimicking renal colic could be found by ultrasonography at least in one study with the same accuracy as MDCT [64].

Some limitations and biases of our meta-analysis should be taken into account. We have no registered a protocol of the systematic review on a database such as PROSPERO. We included some retrospective studies because of the good data quality. Heterogeneity among studies may represent a potential source of bias in a meta-analysis. This heterogeneity is likely to arise through baseline differences among patients in the included studies (Table 1), or diversity in methodological aspects between different studies (Table 2). Unfortunately, we detected a significant heterogeneity in our meta-analysis. We believe that, beyond the various microhematuria tests (urinalysis vs dipstick), the most important source of heterogeneity could be the different definitions of microhematuria (Table 2). Finally, we found presence of publication bias.

In conclusion, microhematuria searched with urine dipstick showed higher diagnostic sensitivity and should be used in this setting as a “gold standard”; it is needed to calculate the STONE score, which can help to identify patients with decreased likelihood of a differential diagnosis, reducing costs and radiation exposure of MDCT. Finally, the concomitant use of ultrasound could increase the specificity till 98% by hydronephrosis, identify patients requiring urological intervention and help to find alternative diagnosis in each risk group. Especially for searching differential diagnosis with ultrasound in patients with suspected renal colic, further studies should be undertaken. Larger prospective multicenter validation study of the STONE score could provide more definitive evidence.

## Supplementary information

**Additional file 1 Supplemental figure 1.** Overall quality assessment of the studies included in the systematic review according to QUADAS-2 tool.

**Additional file 2 Appendix 1.** Search strategy used for PubMed/MEDLINE and Cochrane Central Register of Controlled Trials (CENTRAL).

## Data Availability

Not applicable.

## Abbreviations

MDCT: multi-detector computed tomography
QUADAS: Quality Assessment of Diagnostic Accuracy Studies
CI: Confidence interval
PRISMA: Preferred Reporting Items for Systematic Reviews and Meta-Analyses
BUN: Blood urea nitrogen
ACEP: American College of Emergency Physicians
IQR: Interquartile range
CT: Computed tomography
