# Supplementary figures and images for: Prevalence of microhematuria in renal colic and urolithiasis: a systematic review and meta-analysis
Source: BMC Urol. 2020 Aug 8;20:119. doi: 10.1186/s12894-020-00690-7 (PMC7414650; doi:10.1186/s12894-020-00690-7)

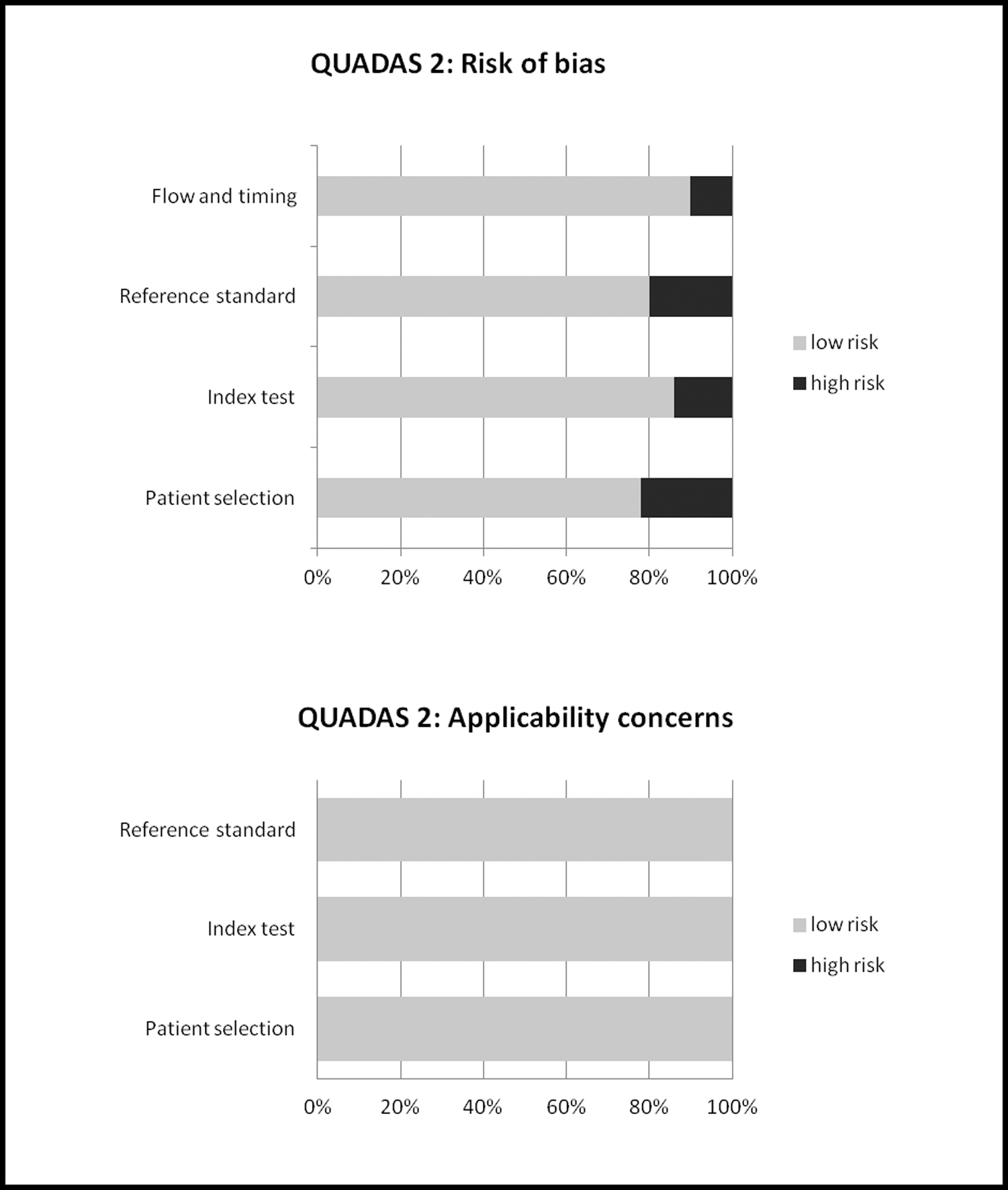

Supplement: Supplementary file 1 — Additional file 1 Supplemental figure 1. Overall quality assessment of the studies included in the systematic review according to QUADAS-2 tool. [file 12894_2020_690_MOESM1_ESM.tif]
