# Supplementary material for: Prevalence of microhematuria in renal colic and urolithiasis: a systematic review and meta-analysis
Source: BMC Urol. 2020 Aug 8;20:119. doi: 10.1186/s12894-020-00690-7 (PMC7414650; doi:10.1186/s12894-020-00690-7)
Supplement: Supplementary file 2 — Additional file 2 Appendix 1. Search strategy used for PubMed/MEDLINE and Cochrane Central Register of Controlled Trials (CENTRAL). [file 12894_2020_690_MOESM2_ESM.docx]

**Appendix 1**

**Search strategy used for PubMed/MEDLINE and Cochrane Central Register of Controlled Trials (CENTRAL)**

(“renal colic*” OR “ureteric colic*” OR “ureteral colic*” OR “painful urolithiasis” OR ((pain OR symptom*) AND (urolithiasis OR ureterolithiasis OR stone* OR nephrolithiasis OR calculi))) AND (microhematuria OR hematuria OR haematuria OR ((microscopic) AND ((hematuria) OR (haematuria))) OR urinalysis OR urinalyses OR ((urine) AND ((analys*) OR (test) OR (tests) OR (testing))))
